# Risk Factors for CMV and BK Infections in an Elderly Veteran Population Following Kidney Transplantation: Implications for Immunosuppression Induction and Management

**DOI:** 10.3390/biomedicines11113060

**Published:** 2023-11-15

**Authors:** Anne Thorndyke, Cara Joyce, Manpreet Samra, Laura Cotiguala, Christine Trotter, Oswaldo Aguirre, W. James Chon, Rupinder Sodhi, Reynold I. Lopez-Soler

**Affiliations:** 1 Department of Pharmacy, Edward Hines VA Jr. Hospital Hines, Hines, IL 60141, USA; anne.thorndyke@va.gov (A.T.); laura.cotiguala@va.gov (L.C.); 2 Department of Medicine, Stritch School of Medicine, Maywood, IL 60153, USA; cjoyce6@luc.edu (C.J.); woojin.chon@va.gov (W.J.C.); rupinder.sodhi@lumc.edu (R.S.); 3 Department of Medicine, Edward Hines VA Jr. Hospital Hines, Hines, IL 60141, USA; manpreet.samra@va.gov; 4 Section of Transplantation, Edward Hines VA Jr. Hospital Hines, Hines, IL 60141, USA; christine.trotter@va.gov (C.T.); oswaldo.aguirre@va.gov (O.A.); 5 Department of Surgery, Division of Intra-Abdominal Transplantation, Stritch School of Medicine, Maywood, IL 60153, USA

**Keywords:** kidney transplant, CMV, BK, co-infection

## Abstract

Cytomegalovirus (CMV) and BK Polyomavirus (BKPyV) are the most common opportunistic pathogens following kidney transplantation. We evaluated 102 patients with a median age of 63 at Edward Hines VA Hospital from November 2020 to December 2022. Our primary interest was the incidence of CMV and BKPyV infections, as well as CMV and BKPyV coinfection. Secondary interests included time to infection, rejection, and graft and patient survival. There were no statistically significant differences in patient age, donor age, race, transplant type, incidence of delayed graft function, or induction in both cohorts (any infection (N = 46) vs. those without (N = 56)). There was a 36% (37/102) incidence of CMV, a 17.6% (18/102) of BKPyV and an 8.8% (9/102) incidence of coinfection. There was a decreased incidence of CMV infection in Basiliximab induction versus antithymocyte globulin (21% and 43%). CMV risk status had no effect on the incidence of CMV infection following transplant. African American recipients had a lower incidence of BKPyV infection (12% vs. 39%), yet a higher incidence was observed in those with high cPRA (50% vs. 14%). Most CMV and/or BKPyV infections occurred within the first six months post-transplant (54%). Immunosuppression management of the elderly should continually be evaluated to reduce opportunistic infections post-transplant.

## 1. Introduction

Kidney transplantation is a life-saving treatment for individuals with end-stage kidney disease, offering an improved quality of life and long-term survival. However, the success of transplantation is often challenged by complications, including viral infections. Among these, co-infection with cytomegalovirus (CMV) and BK polyomavirus (BKPyV) has gained increasing recognition as a significant clinical concern in kidney transplant recipients. CMV, a member of the herpesvirus family, and BKPyV, a polyomavirus, are ubiquitous viruses that can reactivate in immunocompromised individuals. Both viruses can cause significant morbidity and impact graft and patient outcomes following kidney transplantation. While each virus possesses distinct characteristics and clinical manifestations, their co-infection poses unique challenges and warrants careful attention in transplant medicine. The simultaneous presence of these two viruses can lead to intricate interactions that have the potential to affect graft function, increase the risk of rejection, and impact long-term transplant outcomes.

Infections with either BKPyV or CMV have been linked to immunosuppressant use, episodes of acute rejection, age, and pancreas transplantation [1,2,3,4,5]. The degree of immunosuppression has been described as the main risk factor associated with the development of BKPyV. Additionally, risk increases with advancing age. Elderly patients have alterations in their absorption and elimination of immunosuppressants. This can alter the net level of immunosuppression and drug concentrations [6]. Specifically, the use of lymphocyte-depleting agents, tacrolimus and mycophenolate, compared with cyclosporine, are factors that have been associated with an increased risk of developing a BKPyV infection [7]. Other risk factors that have been identified are the number of HLA mismatches, cold ischemia time, previous rejection, male sex, and age [7]. CMV infection has also been associated with multiple factors. Specifically, donor and recipient mismatching (D+/R−) has consistently been shown to be the most prevalent risk factor for CMV infection [5,6,7,8]. However, other risk factors have also been described such as older age, lymphocyte depletion during the induction of immunosuppression, and episodes of rejection [5,6,7,8].

There have not been many studies or much consensus on the risks, presentation, and outcomes for CMV and BKPyV co-infection. Previous studies have shown that hypertension, hyperglycemia, proteinuria, elevated tacrolimus, and MMF dosing were independent risk factors for co-infection, but there was no association with older age [9]. This was not replicated in other studies where age was a risk factor for BKPyV infection, CMV infection, and co-infection [10,11,12]. In fact, other studies have shown that age, acute rejection, diabetes, and nephrostomy tube requirement, but not immunosuppressant use, were risk factors for co-infection [13]. Immune system senescence as well as an over-responsiveness to immunosuppression could also be a causative agent for these post-transplant infections [14]. However, infection with one virus can predispose a patient to co-infection with the other [8,13,15]. While some studies have suggested that each virus might be a risk factor for co-infection, others have found that CMV DNAemia may indirectly protect against subsequent BKPyV DNAemia [15,16,17]. Conversely, other investigators have reported that the management of BKPyV infection may be protective against developing CMV infection [18]. In addition, 36% of patients with BKPyV DNAemia developed CMV infections, and conversely 25% of CMV DNAemic patients developed BKPyV infections, while 17% were diagnosed with both infections simultaneously [12]. In aggregate, these studies have shown a cumulative incidence, between 7 and 12%, for co-infection 1–5 years post transplantation [12].

All studies showed that there were detrimental effects on graft function after transplantation, in the setting of co-infection [12,16]. However, the data are not clear on the effects of the association between co-infection on patient and graft survival [12,16].

The aim of this study was to evaluate the incidence of CMV and BKPyV infections, as well as CMV and BKPyV co-infection, and their impact on graft and patient survival in an elderly veteran population.

## 2. Materials and Methods

This was a retrospective single center study that included 102 patients transplanted at Edward Hines VA Hospital between November 2020 and April 2022. All patients transplanted during that time were included in this study. All kidney recipients were followed for at least six months post-transplantation and data were retrospectively recorded. The demographic characteristics of the patients were recorded at the time of kidney transplantation. Induction therapy was chosen according to center protocol, which included the patient’s immunologic risk. Part of their immunologic evaluation included age; of those 65 or older, 24/52 (46%) were induced with basiliximab, and 3/50 patients (26%) under 65 were induced with basiliximab. The remaining patients received induction with anti-thymocyte globulin. Primarily dual immunosuppression consisted of tacrolimus and mycophenolate with a rapid prednisone taper by post-operative day (POD) 6. Patients that were of high immunologic risk or on prednisone pre-transplant continued their treatment as part of a triple drug immunosuppression regimen.

### 2.1. Monitoring and Definitions

Patients were screened via immunoglobulin assay for prior CMV infection during their pre-transplant evaluation. The donor’s CMV IgG status was also collected from UNOS.

Renally adjusted prophylactic valganciclovir was given to moderate risk (D+/R+) patients for 90 days and for 180 days to patients that were deemed high risk (D+/R−).

Viral load lab testing for BKPyV was monitored in all patients via real-time quantitative nucleic acid amplification testing (QNAT) blood PCR, sent monthly starting at POD28. Results were recorded in copies per milliliter with a lower limit of detection of 500. Viral load lab testing for CMV QNAT were recorded in international units per milliliter with a lower limit of detection of 200, which started monthly after valganciclovir prophylaxis had been completed.

If patients developed CMV DNAemia, they were screened for symptoms and evaluation for treatment Patients were treated if their PCR was >500 IU/mL, regardless of symptoms. They were also treated if their PCR was <500 IU/mL with symptoms consistent with CMV disease or end-organ disease.

CMV DNAemia was defined in accordance with the third international consensus guidelines as evidence of CMV replication regardless of symptoms; defined either via virus or viral protein (antigens) detection or nucleic acid in any body fluid or tissue specimen [19]. CMV disease: evidence of CMV infection with attributable symptoms. CMV disease was further categorized as a viral syndrome (requiring 2 or more of the following: fever, malaise, leukopenia, and/or thrombocytopenia), or as tissue-invasive (“end organ”) disease [19].

If patients developed BKPyV replication, repeat testing in addition to standard protocol was determined by the attending physician. Recipients with confirmed or presumed BK virus were managed via a net reduction in their immunosuppression.

Presumed BKPyV-associated nephropathy (BKVAN) was associated with >10^4^ copies/mL seen in PCR tests for at least four weeks, or confirmed BKVAN via biopsy.

Coinfection was defined as patients that experienced both CMV DNAemia and BKPyV DNAemia simultaneously, or at different time points, that required an adjustment in their immunosuppression or treatment for CMV infection.

Only biopsy-proven rejections were considered relevant to our study. Kidney biopsy was performed based on the physcician’s discretion, that included an increase in serum creatinine, the development of donor specific antibodies, new proteinuria, or an inability to clear viral infections. Rejections were determined based on histological biopsy based off BANFF classification [20].

Graft failure was defined as a permanent return to hemodialysis.

### 2.2. Statistical Analyses

Charts were reviewed through the Computerized Patient Record System (CPRS), and data were collected using Microsoft Excel^®^ (version 2302). Patient characteristics were summarized overall and by type of infection. The rates of each infection type (CMV, BKPYV, both infections) were calculated according to patient characteristics, and hazard ratios with 95% profile-likelihood confidence intervals were estimated in separate univariable Cox proportional hazards regression models of the time to infection. Firth’s adjustment was applied for conditions of monotone likelihood. Analyses were performed using SAS 9.4 (SAS Institute, Cary, NC, USA).

## 3. Results

### 3.1. Patient Demographics

Between November 2020 and December 2022, 102 patients received a kidney transplant. The overall median follow-up time was 14 months (95% CI: 11–18 months). The mean age was 63 ± 9, nearly two-thirds of patients were African American (*n* = 67, 65.6%), and 26.5% were White (*n* = 27). Diabetes (*n* = 46, 45.1%) and hypertension (*n* = 26, 25.5%) were the most common causes of end-stage kidney disease. Most received brain-dead, deceased donor transplants (*n* = 71, 69.6%) with 27.5% DCD (*n* = 28) and 3% (*n* = 3) from living donors. The risk factors evaluated included a history of hepatitis C (*n* = 29, 28.4%), hepatitis B (*n* = 9, 8.8%), their immunologic risk (*n* = 10, 9.8%), and cPRA (*n* = 8 positive, 7.8%). The median KDPI was 53 (interquartile range: 41–75) and 30.4% (*n* = 31) of patients had delayed graft function following transplantation (Table 1).

CMV DNAemia was seen in 37 patients, with 22.2% presenting with CMV syndrome and 1 patient with CMV gastritis. No statistical difference in induction agent or dosing was seen between groups, yet 21% of patients induced with basiliximab developed CMV, whereas it was 41.9% induced with anti-thymocyte globulin < 4 mg/kg, and 44.7% induced with >4 mg/kg. There were no other patient factors that increased the risk for CMV development after transplant. All patients except one were on mycophenolate as the antimetabolite at the time of CMV DNAemia, and only three returned to full-dose mycophenolate after clearance of the virus. Other strategies included the continued reduction of mycophenolate dosage (N = 20), a transition to azathioprine (N = 2), or a transition to an alternative agent such as an mTOR or prednisone (N = 12) (Table 2).

BKPyV DNAemia was seen in 18 patients, with 16.6% presumed BKVAN and 11% with biopsy-proven BKVAN. For patients with BKVAN, 60% also had a CMV co-infection. A higher BKPyV incidence was seen in patients that had high immunologic risk (50% compared to 14.1%). Being of the African American race was seen as protective against the development of BKPyV. The management of BKPyV was a reduction in immunosuppression, with two patients requiring adjunctive therapies of leflunomide (N = 2) and IVIG (N = 1) (Table 3).

### 3.2. Co-Infection

Only one patient over the age of 65 experienced co-infection, the remaining eight patients were under the age of 65 (*p* = 0.03).

The management of co-infection included a reduction of mycophenolate and tacrolimus troughs. For those that cleared both infections, only one patient was titrated back up to full-dose mycophenolate. The remainder of patients were kept on half dose (N = 3), transitioned to azathioprine (N = 3), or transitioned to an mTOR or prednisone (N = 3, Table 4).

**Figure 1:**
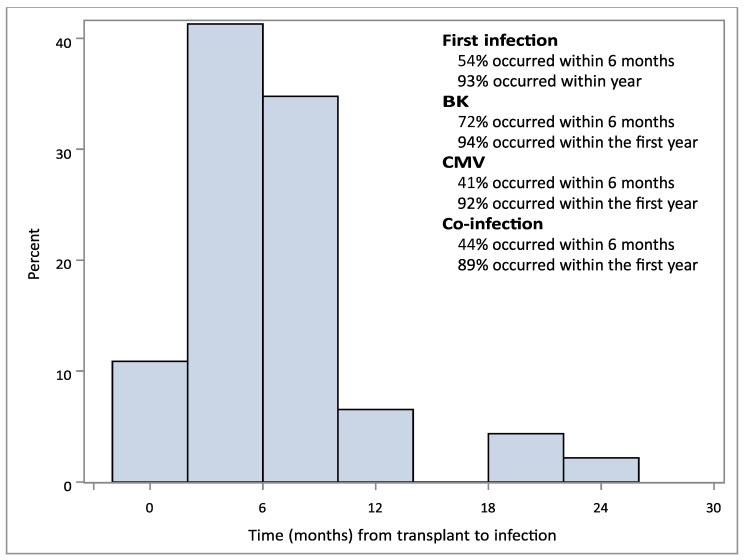
Time to infection/co-infection following transplant. Time to infection with either CMV alone, BK alone, or CMV/BK co-infection was determined in months.

Overall, 54% of those with any infection experienced it within the first six months following transplantation and 93% within the first year (Figure 1). CMV and CMV/BKV co-infection followed a similar pattern, with most of the infections occurring within the first year (92% vs. 89%, Figure 1). BK infection tended to occur earlier, with 72% of infections occurring within the first 6 months compared to 41% for CMV (Figure 1).

### 3.3. Transplant Outcomes

The incidence of acute cellular rejection was low across the cohort, at 7.8%, with low rates of ABMR (antibody-mediated rejection) at 1%. ACR (acute cellular rejection) in the co-infected group was 33.3%, which was higher than for patients that experienced neither infection, for whom ACR was at 5.3% (Table 1).

Allograft survival was high, with only three graft failures noted in the follow-up period; two patients were not diagnosed with either BKV or CMV infection, while one patient had CMV DNAemia alone. Over all 1-year patient survival was 96.1%. Of the patients that died, one patient had no evidence of infection, and three patients had either CMV or BKPyV. None of the patients with co-infection died during the study period.

## 4. Discussion

Our study focused on the risks and management of CMV and BK co-infection following a kidney transplant in an elderly veteran population. Interestingly, there was some agreement with previously published results, but we did notice some differences. Therefore, our data point to the need for special considerations in older transplant recipients to minimize the development of BKV and CMV co-infection.

The median age of our study population was 63. This allows us to examine BKV/CMV co-infection in an elderly population for the first time. We showed that an age greater than 65 years appeared to be protective for the development of co-infection. This goes against some previous reports, in which elderly patients were at a particularly higher risk for co-infection [10,11,12]. A possible explanation for this finding may be our center’s practice of avoiding lymphocyte-depleting therapy for induction in elderly patients with low immunologic risk, irrespective of race. Our practice is to use basiliximab in this patient population, which we found to result in much lower risk for BK/CMV co-infection (Table 1). Interestingly, while our data showed that advanced age was not a predictor for co-infection, CMV and BKPyV mono-infection was still prevalent. For patients older than 65 years, 47.9% experienced either CMV (33.4%) or BKPyV DNAemia (15.6%). It is possible that the risk for co-infection is much more strongly linked to the degree of immunosuppression, when compared to the risk for single viral infections in the elderly, particularly with the use of lymphocyte-depleting agents for induction. In addition, most co-infections occurred later after transplant, compared to BK infections, and appeared to follow the temporal pattern of CMV DNAemia (Figure 1; 72% vs. 44% in the first 6 months). The association between CMV and BKV infection and CMV/BKV co-infection will require additional investigation.

There were patient-level characteristics that were associated with an increased co-infection risk. Our data, in agreement with previous data, do show that increasing immunosuppression leads to an increasing risk of co-infection. Patients with increased immunologic risk were found to be at higher risk for co-infection. Accordingly, our data shows that cPRA > 0, and DCD kidney transplantation resulted in an increased risk for co-infection. This is likely due to the need for stronger induction therapies (i.e., anti-thymocyte globulin vs. basiliximab) and the stronger maintenance of immunosuppression. Interestingly, we did not detect a significant difference in the rates of co-infection with the use of either standard-dose (>4 mg/kg) or lower-dose (<4 mg/kg) thymoglobulin. This indicates that lymphocyte depletion is an independent risk factor for co-infection. These higher-risk patients were also observed to have increased prevalence of ACR within the first year following transplant. Therefore, advanced age was not protective against co-infection in the setting of an increased immunologic risk. Other trials have shown that mTOR use may be protective against co-infection, but no data have demonstrated this [21,22,23,24,25]. It will be intriguing to see if a transition from Tacrolimus-based immunosuppression maintenance (our practice), to mTOR-based, will change the risk profile for co-infection in elderly transplant recipients.

Fortunately, we did not find any effects on patient and graft survival when comparing BK and CMV mono-infection to BK/CMV co-infection. However, there was a more delayed graft function in the co-infection group. This increase in DGF in the co-infection group likely represents yet another risk factor for co-infection, as these patients tended to have longer lengths of hospital stay, leading to a increased recovery period post-transplant. The longer convalescence time for elderly patients potentially results in a longer immune system recovery following the transplant. Finally, long-term outcomes did not appear to be affected by co-infection. A weakness of our study is that it comprises a 2.5-year study period and therefore may not yet capture the effects of co-infection on long-term graft and patient survival.

Management of these infections consisted of immunosuppression reduction with excellent clinical results. However, most patients were not titrated back up to their same level of immunosuppression maintenance as prior to their co-infection. The long-term management of immunosuppression following CMV and BK co-infection could be an area for future exploration.

## 5. Conclusions

Our data reveal that there is a decreased incidence of CMV infection in both DBD and DCD donor organs, as well as with the use of Basiliximab induction compared to thymoglobulin. CMV risk status had no effect on the incidence of CMV infection following transplant. African American recipients had a lower incidence of BKPyV infection but a higher incidence was observed with increasing sensitization. Finally, we found that most CMV and/or BKPyV infections in elderly recipients occurred within the first six months following transplant. Therefore, the immunosuppression management of the elderly should continually be evaluated to reduce opportunistic infections post-transplant. CMV and BKPyV co-infection poses a significant challenge in kidney transplant recipients, affecting graft function, patient outcomes, and long-term survival. Understanding the complexities of co-infection will be crucial for the timely diagnosis, appropriate management, and development of targeted interventions.

## Figures and Tables

**Table 1:** Patient characteristics by infection.

	Overall N = 102	CMV and BKPyV N = 9	CMV Only N = 28	BKPyV Only N = 9	No Infection N = 56
Age, mean (SD)	63 (9)	59 (7)	63 (9)	67 (7)	63 (10)
Race/ethnicity, *n* (%)					
African American	67 (65.7)	5 (55.6)	22 (78.6)	3 (33.3)	37 (66.1)
White	27 (26.5)	1 (11.1)	6 (21.4)	5 (55.6)	15 (26.8)
Hispanic	4 (3.9)	2 (22.2)	0 (0.0)	0 (0.0)	2 (3.6)
Other	4 (3.9)	1 (11.1)	0 (0.0)	1 (11.1)	2 (3.6)
Cause of ESRD, *n* (%)					
Hypertension	26 (25.5)	0 (0.0)	13 (46.4)	2 (22.2)	11 (19.6)
Diabetes	46 (45.1)	7 (77.8)	11 (39.3)	3 (33.3)	25 (44.6)
Other	30 (29.4)	2 (22.2)	4 (14.3)	4 (44.4)	20 (35.7)
Transplant type, *n* (%)					
DCD	28 (27.5)	5 (55.6)	8 (28.6)	1 (11.1)	14 (25.0)
DDKT	71 (69.6)	4 (44.4)	18 (64.3)	8 (88.9)	41 (73.2)
LRKT	2 (2.0)	0 (0.0)	1 (3.6)	0 (0.0)	1 (1.8)
LUKT	1 (1.0)	0 (0.0)	1 (3.6)	0 (0.0)	0 (0.0)
History of hepatitis C, *n* (%)	29 (28.4)	3 (33.3)	8 (28.6)	4 (44.4)	14 (25.0)
History of hepatitis B, *n* (%)	9 (8.8)	0 (0.0)	3 (10.7)	1 (11.1)	5 (8.9)
Immunologic risk, *n* (%)	10 (9.8)	2 (22.2)	1 (3.6)	3 (33.3)	4 (7.1)
CMV risk, *n* (%)					
1	6 (5.9)	0 (0.0)	0 (0.0)	1 (11.1)	5 (8.9)
2	48 (47.1)	5 (55.6)	12 (42.9)	4 (44.4)	27 (48.2)
3	48 (47.1)	4 (44.4)	16 (57.1)	4 (44.4)	24 (42.9)
cPRA > 0, *n* (%)	8 (7.8)	3 (33.3)	1 (3.6)	1 (11.1)	3 (5.4)
Donor age, mean (SD)	42 (13)	42 (11)	44 (12)	39 (14)	42 (13)
KDPI, median (IQR)	53 (41–75)	57 (49–80)	60 (42–84)	43 (42–67)	51 (35–68)
Induction, *n* (%)					
<4 mg/kg	31 (30.4)	4 (44.4)	9 (32.1)	1 (11.1)	17 (30.4)
>4 mg/kg	38 (37.3)	4 (44.4)	13 (46.4)	3 (33.3)	18 (32.1)
Basiliximab	33 (32.4)	1 (11.1)	6 (21.4)	5 (55.6)	21 (37.5)
Delayed graft function, *n* (%)	31 (30.4)	4 (44.4)	5 (17.9)	3 (33.3)	19 (33.9)
Death, *n* (%)	2 (2.0)	0 (0.0)	1 (3.6)	0 (0.0)	1 (1.8)

SD—standard deviation; IQR—interquartile range; ESRD—end stage kidney disease; cPRA—Calculated Panel Reactive Antibody; KDPI—Kidney Donor Profile Index; DCD—donation after circulatory death; DDKT—deceased donor kidney transplantation; LRKT—living related kidney transplantation; LUKT—living unrelated kidney transplantation.

**Table 2:** Univariable associations between patient characteristics and CMV.

	N	*n* (%) with CMV	Hazard Ratio (95% Confidence Interval)	*p*-Value
Overall	102	37 (36.3)	--	
Age, 5-year increase	--	--	0.95 (0.80–1.13)	0.52
Age				
<65	54	21 (38.9)	1 (ref)	0.62
≥65	48	16 (33.3)	0.85 (0.44–1.62)	
Race/ethnicity				
African American	67	27 (40.3)	1 (ref)	0.58
White	27	7 (25.9)	0.66 (0.27–1.41)	
Other	8	3 (37.5)	0.81 (0.22–2.16)	
Cause of ESRD				
Diabetes	46	18 (39.1)	1 (ref)	0.11
Hypertension	26	13 (50.0)	1.30 (0.63–2.60)	
Other	30	6 (20.0)	0.50 (0.19–1.16)	
Transplant type				
DDKT	28	13 (46.4)	1 (ref)	0.02
DCD	71	22 (31.0)	1.62 (0.80–3.14)	
LKT	3	2 (66.7)	5.07 (1.01–16.3)	
History of hepatitis C				
No	73	26 (35.6)	1 (ref)	0.89
Yes	29	11 (37.9)	0.95 (0.46–1.87)	
History of hepatitis B				
No	93	34 (36.6)	1 (ref)	0.97
Yes	9	3 (33.3)	1.02 (0.28–2.69)	
Immunologic risk				
No	92	34 (37.0)	1 (ref)	0.96
Yes	10	3 (30.0)	0.97 (0.26–2.57)	
CMV risk				
1	6	0 (0.0)	1 (ref)	0.28
2	48	17 (35.4)		
3	48	20 (41.7)	1.41 (0.75–2.71)	
cPRA > 0				
0	94	33 (35.1)	1 (ref)	0.38
>0	8	4 (50.0)	1.54 (0.50–3.73)	
Donor age, 5-year increase	--	--	1.05 (0.93–1.19)	0.44
Donor age				
<40	45	15 (33.3)	1 (ref)	0.56
≥40	57	22 (38.6)	1.21 (0.64–2.36)	
KDPI, 10-point increase	--	--	1.10 (0.96–1.27)	0.17
KDPI				
<50	45	13 (28.9)	1 (ref)	0.32
≥50	54	22 (40.7)	1.40 (0.72–2.83)	
Induction				
<4 mg/kg	31	13 (41.9)	1 (ref)	0.18
>4 mg/kg	38	17 (44.7)	1.06 (0.52–2.20)	
Basiliximab	33	7 (21.2)	0.50 (0.19–1.20)	
Delayed graft function				
No	71	28 (39.4)	1 (ref)	0.44
Yes	31	9 (29.0)	0.75 (0.34–1.52)	

**Table 3:** Univariable associations between patient characteristics and BKPyV.

	N	*n* (%) with BKPyV	Hazard Ratio (95% Confidence Interval)	*p*-Value
Overall	102	18 (17.6)	--	
Age, 5-year increase	--	--	1.04 (0.81–1.36)	0.77
Age				
<65	54	11 (20.4)	1 (ref)	0.44
≥65	48	7 (14.6)	0.69 (0.26–1.76)	
Race/ethnicity				
African American	67	8 (11.9)	1 (ref)	0.03
White	27	6 (22.2)	2.05 (0.67–5.91)	
Other	8	4 (50.0)	4.43 (1.18–14.1)	
Cause of ESRD				
Diabetes	46	10 (21.7)	1 (ref)	0.26
Hypertension	26	2 (7.7)	0.30 (0.05–1.16)	
Other	30	6 (20.0)	0.91 (0.31–2.46)	
Transplant type				
DDKT	71	12 (16.9)	1 (ref)	
DCD	28	6 (21.4)	1.21 (0.46–3.16)	0.88
LKT	3	0 (0.0)	--	
History of hepatitis C				
No	73	11 (15.1)	1 (ref)	0.32
Yes	29	7 (24.1)	1.62 (0.59–4.13)	
History of hepatitis B				
No	93	17 (18.3)	1 (ref)	0.82
Yes	9	1 (11.1)	0.83 (0.09–3.27)	
Immunologic risk				
No	92	13 (14.1)	1 (ref)	0.004
Yes	10	5 (50.0)	4.13 (1.32–11.0)	
CMV risk				
1	6	1 (16.7)	1 (ref)	0.96
2	48	9 (18.8)	0.99 (0.19–18.2)	
3	48	8 (16.7)	0.86 (0.16–15.9)	
cPRA > 0				
0	94	14 (14.9)	1 (ref)	0.005
>0	8	4 (50.0)	4.34 (1.23–12.14)	
Donor age, 5-year increase	--	--	0.94 (0.78–1.13)	0.53
Donor age				
<40	45	9 (20.0)	1 (ref)	0.62
≥40	57	9 (15.8)	0.79 (0.31–2.03)	
KDPI, 10-point increase	--	--	1.03 (0.85–1.26)	0.75
KDPI				
<50	45	8 (17.8)	1 (ref)	0.94
≥50	54	10 (18.5)	1.04 (0.41–2.71)	
Induction				
<4 mg/kg	31	5 (16.1)	1 (ref)	0.97
>4 mg/kg	38	7 (18.4)	1.03 (0.33–3.50)	
Basiliximab	33	6 (18.2)	1.14 (0.34–3.97)	
Delayed graft function				
No	71	11 (15.5)	1 (ref)	0.39
Yes	31	7 (22.6)	1.51 (0.56–3.84)	

**Table 4:** Univariable associations between patient characteristics and BKPyV/CMV coinfection.

	N	% with Both Infections	Hazard Ratio (95% Confidence Interval)	*p*-Value
Overall	102	9 (8.8)	--	
Age, 5-year increase	--	--	0.84 (0.60–1.18)	0.28
Age				
<65	54	8 (14.8)	1 (ref)	0.03
≥65	48	1 (2.1)	0.19 (0.02–0.85)	
Race/ethnicity				
African American	67	5 (7.5)	1 (ref)	0.006
White	27	1 (3.7)	0.72 (0.07–3.59)	
Other	8	3 (37.5)	5.09 (1.18–19.1)	
Cause of ESRD				
Diabetes	46	7 (15.2)	1 (ref)	0.08
Hypertension	26	0 (0.0)	--	
Other	30	2 (6.7)	0.51 (0.09–1.89)	
Transplant type				
DDKT	72	7 (9.7)	1 (ref)	
DCD	30	2 (6.7)	3.11 (0.88–11.6)	0.12
LKT	3	0 (0.0)	--	
History of hepatitis C				
No	73	6 (8.2)	1 (ref)	0.74
Yes	29	3 (10.3)	1.24 (0.29–4.41)	
History of hepatitis B				
No	93	9 (9.7)	1 (ref)	0.63
Yes	9	0 (0.0)	--	
Immunologic risk				
No	92	7 (7.6)	1 (ref)	0.05
Yes	10	2 (20.0)	3.52 (0.65–13.3)	
CMV risk				
1	6	0 (0.0)	1 (ref)	0.82
2	48	5 (10.4)		
3	48	4 (8.3)	0.87 (0.23–3.07)	
cPRA > 0				
0	94	6 (6.4)	1 (ref)	<0.001
>0	8	3 (37.5)	7.26 (1.72–25.8)	
Donor age, 5-year increase	--	--	1.00 (0.78–1.29)	0.97
Donor age				
<40	45	4 (8.9)	1 (ref)	0.98
≥40	57	5 (8.8)	1.01 (0.29–3.78)	
KDPI, 10-point increase	--	--	1.06 (0.80–1.40)	0.67
KDPI				
<50	45	3 (6.7)	1 (ref)	0.49
≥50	54	6 (11.1)	1.54 (0.44–6.48)	
Induction				
<4 mg/kg	31	4 (12.9)	1 (ref)	0.40
>4 mg/kg	38	4 (10.5)	0.73 (0.19–2.87)	
Basiliximab	33	1 (3.0)	0.32 (0.03–1.74)	
Delayed graft function				
No	71	5 (7.0)	1 (ref)	0.25
Yes	31	4 (12.9)	1.97 (0.53–6.96)	

## Data Availability

The data underlying this article are available in the article and online. The data underlying this article will be shared on reasonable request to the corresponding author.
